# Corporate Digital Transformation and Green Innovation: A Quasi-Natural Experiment from Integration of Informatization and Industrialization in China

**DOI:** 10.3390/ijerph192013606

**Published:** 2022-10-20

**Authors:** Qincheng Zhang, Mingzeng Yang, Shanshan Lv

**Affiliations:** 1 School of Accountancy, Shandong University of Finance and Economics, Jinan 250014, China; 2 College of Foreign Languages, Shandong Agricultural University, Tai’an 271018, China; 3 Graduate School, Our Lady of Fatima University, Manila 1440, Philippines

**Keywords:** digital transformation, green innovation, Integration of Informatization and Industrialization, DID

## Abstract

In the era of the digital economy, the rise and application of digital technologies have led to a series of systematic changes and disruptive innovations within enterprises. Based on the quasi-natural experiment of “Integration of Informatization and Industrialization”, this paper examines the economic consequences of digital transformation from the standpoint of corporate green innovation, utilizing China’s listed manufacturing firms as the research object. Using the DID model, it is discovered that through the implementation of corporate digital transformation, the output of green innovation increases significantly. The conclusions are still robust when using the parallel trend test, PSM-DID, placebo test, and the test of deleting the sample entering the pilot in the current year. Extended analyses find that corporate digital transformation has a greater effect on green innovation in regions with weaker digital economy, in industries with less rivalry, and in firms with larger size. The conclusions of this paper not only advance research on digital transformation and its economic consequences, but also provides theoretical proof and practical insights for advancing corporate digital transformation and enhancing the green development system.

## 1. Introduction

Environmental problems, such as climate change caused by global warming, are considered a persistent and extensive hazard that poses an enormous threat to human life and sustainable development [1]. In most cases, however, environmental deterioration and ecological harm result from the production and manufacturing activities of enterprises [2]. As environmental issues such as global warming and the oil crisis gain prominence, society’s environmental protection obligations for enterprises become increasingly stringent. Given this, enterprises have opted for green innovation as an effective method to achieve cleaner production and improved competitiveness [3] because green innovation may drastically reduce the negative environmental impact of production and operations [4].

In the era of the digital economy, digital technologies such as artificial intelligence, blockchain, cloud computing, big data, and the Internet of Things (IoT) are being applied broadly and intensively to the real economy, forcing enterprises to explore transformational pathways. The rapid expansion of the digital economy presents excellent opportunities for enterprises’ breakthrough innovation through the use of digital technologies, which would reshape enterprises’ production and operation models and play an increasingly crucial role in improving the efficiency of resource utilization [5]. Existing research indicates that institutional pressure [6], market demand [7], innovation capacity [8], and organizational factors [9] are vital drivers of corporate green innovation. Despite this, there is a paucity of research addressing how digital transformation fosters green innovation [10], particularly, there is lack of empirical evidence from the micro perspective of enterprises [11]. Consequently, this paper studies the economic consequences of digital transformation from the standpoint of green innovation on the micro perspective of enterprises.

Despite China’s ascending economy in recent years, major environmental degradation has also been brought on by its extensive development model of high investment, high pollution, and high energy-consumption [2]. It is a pressing issue for China to switch from an extensive development model to a green development model driven by total factor productivity [12]. In order to attain “zero CO_2_ emissions” by 2060, the Chinese government has committed to actively implementing the idea of green development, halting the growth of CO_2_ emissions by 2030 and offsetting its own emissions by planting trees, conserving energy, and cutting emissions. Meanwhile, China has always emphasized and promoted the deep integration of information technology with the real economy, with the purpose of seizing opportunities of the new industrial revolution, building new digitally-driven industrial ecologies, and achieving high-quality economic development. In 2013, The Assessment Specification of Industrial Enterprises’ Informatization and Industrialization Integration (GBT23020-2013) was issued by China’s National Standardization Administration Committee, which emphasized strengthening the digitalization of the whole manufacturing process by 23 times and acted as the national criterion for promoting corporate digitalization. In 2014, the pilot project of the Integration of Informatization and Industrialization (IoII) was launched throughout China by the Ministry of Industry and Information Technology (MIIT). Those that met the criteria of the Specification were recognized as pilot enterprises. China’s pilot project of IoII presents a sound quasi-natural experimental setting for determining the economic consequences of corporate digital transformation. On the basis of this theoretical and practical background, by selecting pilot enterprises of IoII as the treatment group and those non-pilot enterprises as the control group, we apply the difference-in-differences (DID) model to explore the causal relationship between corporate digital transformation and green innovation, as well as how the macro external environment, meso industry attributes, and micro enterprise characteristics influence this relationship in order to further pursue the sustainable advantages of green innovation.

This paper makes three contributions. First, the existing research on digital transformation focuses mostly on corporate performance and capital market performance, but less on corporate environmental sustainability. We study the economic consequences of corporate digital transformation from the standpoint of green innovation, hence enriching research on digital transformation and its economic consequences. Then, we analyze and empirically test the impact of corporate digital transformation on environmental sustainability, thus extending research on the impact factors of corporate green innovation. Third, we examine the enhancement function of corporate digital transformation on environmental sustainability, providing empirical evidence through the implementation of the “IoII” policy, as well as a theoretical foundation and practical recommendation for advancing corporate digital transformation and promoting environmental sustainability.

The reminder of the paper is structured as follows: Section 2 reviews the literature on corporate digitalization and green innovation; Section 3 demonstrates the hypotheses of the paper; Section 4 details the research design; Section 5 presents and discusses the primary results and robustness tests; Section 6 provides extended analysis; and Section 7 concludes the paper.

## 2. Literature Review

### 2.1. Economic Consequences of Corporate Digital Transformation

In the era of the digital economy, the deep integration between digital technology and the real economy creates the proliferation of new products, services, and commercial modes, thereby fostering economic development and producing great ecological value. The existing research on the economic consequences of corporate digital transformation is mainly carried out from two aspects: the transformation process and the completion of the transformation.

In the process of digital transformation, the organizational structure, dynamic capabilities, and corporate strategies of enterprises are all affected by the application of digital technologies. (1) In terms of organizational structure, driven by digital technologies, the digitalization transformation has produced disruptive changes in organizational resources and firms’ deep structure [13,14]. Meanwhile, interdependence among corporations has been increasing [15]. (2) In terms of dynamic capabilities, with the deepening of digital transformation, enterprises can rely on digital technology to integrate and build internal and external capabilities to cope with the rapidly changing environment, so as to improve dynamic capabilities and gain competitive advantages [16]. (3) In terms of corporate strategy, digital transformation guides enterprises to reconsider the role of digital technology in formulating their business strategies and breaks the barrier between business and technology to achieve close collaboration [17], which triggers enterprises to adjust their development strategies [18].

Existing research has found that digital transformation can significantly improve business performance. Through digital product innovation, enterprises create new value for customers and then improve performance [19], which is reflected in rapidly capturing consumer demand information, reducing information collection costs, and improving market position. (1) The application of digital technology enables enterprises to capture and respond to market changes more quickly [20], thus realizing rapid iteration and continuous optimization of products. Hansen and Kien (2015) [21] found that the ability to use digital technology to collect feedback information and interact with customers in real time enabled the company to better respond to consumer needs through studying the European sports and fashion retail company Hummel. (2) Digital transformation also enables enterprises to reduce costs [22]. For example, Andal-Ancion et al. [23] found that information technology would cut enterprises’ search costs and generate more effective decisions. (3) Digital transformation improves business performance and market position [24]. Digital technology is the foundation to maintain competitive advantage and create new value for customers [25], and a new source to change the way enterprises create business value [26]. Digital technologies not only improve the efficiency of innovation and research and development and increase the possibility of cross-border integration, but also enrich the way value is created, allowing companies to respond more flexibly to environmental changes and thus achieve more superior performance [27].

In recent years, the impact of digitalization on environmental sustainability has been researched in more depth [28]. According to de Sousa Jabbour et al. [29], digital technologies in Industry 4.0 support environmental sustainability in manufacturing. Costa and Matias [30] discussed that digital transformation builds a sustainable innovation ecosystem through open innovation. Chen et al. [28] indicated that by tracking and optimizing resource consumption, digitization achieves environmental sustainability of manufacturing processes.

From the aforementioned literature, it is evident that scholars have conducted fruitful research on corporate digital transformation and have begun to focus on its impact on environmental sustainability, providing a solid foundation for future research on the environmental benefits of digital transformation. There is a shortage of empirical research regarding how manufacturing enterprises employ digital technologies to become environmentally friendly [11]. This study explores the role of corporate digital transformation in green development from the perspective of corporate green innovation, and deepens the research on the relationship between corporate digital transformation and environmental sustainability.

### 2.2. Influencing Factors of Corporate Green Innovation

Corporate green innovation is a systematic behavior driven by crucial internal and external factors. The existing research on the drivers of green innovation can be roughly divided into two levels: internal and external. From the external perspective of enterprises, Wagner [31] believed that the environmental awareness of stakeholders has a great impact on the green innovation output of enterprises. Schaefer [6] discovered that institutional pressure drives green corporate behavior. According to Horbach [32], government regulation promotes environmental management, energy conservation, emission reduction, noise reduction, and product recycling. Eiadat et al. [33] proposed that market instruments drive green innovation and help enterprises establish incentive structures for the circular economy. According to Lee [34], buyer influence, government cooperation, and the maturity of the green supply chain motivate enterprises to adopt green practices. Demirel and Kesidou [35] believed that environmental legislation and cost-saving stimulate corporate green innovation. In addition, environmental governance [36,37,38], government subsidies [39,40], market demand [7], incentive policies [41,42,43], and FDI [44] are also influencing factors on corporate green innovation. From the internal perspective of enterprises, existing research mainly focuses on enterprises’ innovation capability [8], organizational characteristics [9], managers’ willingness [45], knowledge and technology [46], organizational cooperation [47], green perception ability [48], organizational strategy [49], human resources [50], and other influencing factors.

### 2.3. Impact of Digital Technologies on Green Innovation

In recent years, with the development of digital technology, more and more scholars have begun to pay attention to the impact of digital technology on green innovation. These studies mainly involve digital technologies such as artificial intelligence, big data, cloud computing, and blockchain. First, regarding artificial intelligence, Yang et al. [51] found that manufacturing intelligence has a significant promotion effect on green innovation performance. The reason is that manufacturing intelligence is conducive to “technology promotion effect” and “cost reduction effect”, so as to promote green technology innovation. Su et al. [52] indicated that artificial intelligence empowers green radical innovation of high-tech enterprises. Second, in terms of big data, Waqas et al. [53] found the role of big data analytics in boosting green innovation in the Chinese manufacturing industry. Dong et al. [54] discovered that green innovation acted as a mediator in the relationship between external institutions and competitive advantage. The adoption of big data and predictive analytics positively moderated this mediation effect. Third, in terms of cloud computing, Wang [55] proved that the application of cloud computing had a huge effect on the green investment evaluation system. Fourth, regarding blockchain technology, Chin et al. [56] found that blockchain technology positively affects green innovation performance, while value appropriation capability also mediates the blockchain technology–green innovation relationship in ecosystem-based business models. Polas et al. [57] investigated the role of blockchain technology in green innovation practices and found that blockchain technology mediates the relationship between sustainability orientation and social perception with the adoption of green innovation that employs green energy technology towards a sustainable green economy.

In summary, digitalization is becoming the core strategic direction of global technological change, and digital transformation has evolved into an important path to high-quality economic development. This will inevitably bring about disruptive innovations in corporate management paradigms and even management systems, thus bringing about impacts on corporate green innovation and forcing evolutionary changes in all elements and processes of corporate green innovation. The question of whether digital transformation can promote green innovation in enterprises is thus of interest, but empirical evidence to answer this question is lacking in the literature. Based on this, this paper uses the quasi-natural experimental scenario provided by the gradual implementation of the pilot mechanism of the “Integration of Informatization and Industrialization“ and adopts the difference-in-differences (DID) method to study the impact of digital transformation on enterprise green innovation, which not only helps to enrich the research on the economic consequences of digital transformation and further expand the research on the influencing factors of enterprise green innovation, but also provides a useful reference for deepening the integration of informatization and industrialization and the digital transformation of enterprises, and promoting the high-quality development of enterprises.

## 3. Hypothesis Development

According to existing studies, green innovation requires integrating resource consumption and manufacturing processes, internal and external technical skills, and enhanced information sharing. AI, blockchain, cloud computing, big data, and the Internet of Things provide enterprises with new opportunities for green innovation. Through data mining, information sharing, and knowledge integration, corporate digital transformation may optimize green innovative resources and then enhance green technology innovation.

(1) Data mining. Corporate digital transformation has data mining functions to boost the system’s internal data vitality, and to help enterprises optimize their existing manufacturing process by enhancing energy efficiency and lowering pollutant emissions. From product design to terminal distribution, enterprises have acquired vast volumes of data. Before digital transformation, the efficiency of data processing is low, and enterprises cannot effectively mine the laws implicit in the data. Nevertheless, through the implementation of digital transformation, enterprises are equipped to process enormous, non-standardized, unstructured data with digital technologies, encode and output it as structured and standardized information, and increase information availability.

An intelligent production system is equal to automating data collection, storage, analysis, production, monitoring, and management [58]. Precision sensing technology and an intelligent system enables enterprises to monitor and analyze the production process, instantly complete the traceability and positioning of high-energy consumption links, combined with big data mining to continuously improve the follow-up production process, and provide a reliable basis for green innovation project decisions. Cloud computing also makes data mining economical for enterprises. Therefore, through the implementation of digital transformation, enterprises have more data mining space, better innovation decision-making ability, and deeper R & D project knowledge.

Enterprises can fully utilize this data to make the best production and sales decisions to optimize production and sale processes [59], ensuring that their processing and manufacturing procedures follow environmental laws, and minimizing the negative effects on the environment.

(2) Information sharing. Corporate digital transformation can be capable of accelerating information exchange, promoting the sharing of information related to internal and external environment and resources, so as to motivate enterprises to engage in green technology innovation activities. Traditional enterprise information transfer and communication is inefficient, confined by time and space. However, digital technologies have eliminated barriers to achieving instant transmission of information and instant communication between individuals, hence significantly enhancing communication efficiency.

Internal and external information sharing are included in information sharing. Internal information sharing refers to the transfer and integration of data between organizational divisions [60]. Digital technologies enhance links between employees, departments, and objects. Built by digital technologies such as cloud computing and the Internet of Things, data centers or data center clusters will efficiently collect and store enterprise data, share data via cloud platforms to break down “departmental walls”, eliminate “information silos” within the enterprise, and generate complementary innovations through the effective integration of internal resources [61]. External information sharing stresses corporate contact and collaboration with external market participants. External information sharing is more effective as a result of digital transformation. Digital technologies enable enterprises to communicate more effectively with suppliers, customers, and governments, enabling them to possess external information in real time and facilitating internal and external communication and interaction. This will encourage information sharing to stimulate enterprises’ green innovation [62].

Given this, digital transformation may increase the efficiency of information sharing within enterprises, as well as between internal and external partners, thereby fostering green innovation.

(3) Knowledge integration. Corporate digital transformation may promote the integration of R & D resources and knowledge to effectively accelerate enterprises to carry out green innovation activities. From the perspective of knowledge, green innovation is essentially a complex knowledge activity, involving the creation, integration, and diffusion of knowledge in different technological fields, such as corporate production and pollution reduction. It is challenging to obtain green innovation achievements with experience and knowledge from simply one technological discipline. To master green innovation’s mainstream technology, new ideas, and development trends, enterprises must combine information from several technical sectors, and manage and apply internal and external knowledge. Integration of knowledge is therefore an essential and effective method for manufacturing enterprises to execute green innovation.

Digital technologies help enterprises integrate knowledge. Agostino and Donati [63] assumed that digital technologies may aid enterprises in enhancing exploratory search effects, gaining clear insight into their internal knowledge, and rapidly identifying and integrating external knowledge. Digital capture and intelligent analysis systems accelerate the integration of data resources, hence enhancing the effectiveness of green innovation decisions. Digital transformation links and aggregates data to provide innovation-relevant information [11]. In addition, digital technologies will expand the area for innovation resource allocation, and encourage enterprises, universities, and research institutes to participate in cross-regional and cross-disciplinary collaborative innovation activities. According to Mubarak et al. [64], digital technologies may stimulate open innovation in enterprises, enabling employees to engage in green innovation. Therefore, digital technologies are equipped to facilitate the integration and interchange of R & D resources, as well as the integration and reconfiguration of diverse knowledge elements across multiple technological disciplines, thereby boosting corporate green innovation.

**Figure 1:**
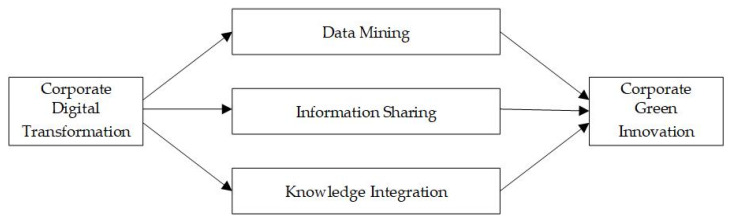
Logic chain of corporate digital transformation affecting green innovation.

In summary, through data mining, information sharing, and knowledge integration, corporate digital transformation may drive green innovation and improve environmental sustainability. The logic chain is shown in Figure 1. Based on the above analysis, this paper proposes the following hypothesis.

**Hypothesis** **1.** *Corporate digitalization has a positive impact on green innovation*.

## 4. Research Design

### 4.1. Regression Models

Considering the various timing of the sample firms’ inclusion into the integration pilot list, we refer to Beck et al. [65] and employ the following difference in differences model with non-synchronous policy shocks to examine the effect of corporate digital transformation on green innovation.

(1) $$GI_{i,t}=\alpha_{0}+\alpha_{1}Digit_{i,t-1}+\partial X_{i,t}+\Sigma Year+\Sigma Industry+\Sigma Firm+\varepsilon_{i,t}$$

### 4.2. Main Variables

#### 4.2.1. Dependent Variable

In this paper, corporate green innovation (*GI_i,t_*) is the dependent variable. Using Pan et al. [2] and Chen et al. [66] as references, we evaluate green innovation based on the number of green patent applications, since application data are more consistent, dependable, and timely than award data. This paper showcases green innovation based on IPC codes in the “Green List of International Patent Classification” of the World Intellectual Property Organization (WIPO). The types of patents include patents for inventions, utility models, and designs. Since design patents are not classified by the IPC, only invention and utility model patents are considered in this paper. Invention patents are more inventive and technical, whereas utility model patents contain a low degree of innovation and only protect the shape and structure of the product. Based on patent categories, three criteria are developed to quantify corporate green innovation. Specifically, the sum of the number of green invention patent applications and green utility model patent applications measures the total amount of green innovation (GI1), the number of green invention patent applications evaluates the quality of green innovation (GI2), and the number of green utility model patent applications serves as a comparative indicator to measure the amount of green innovation (GI3). In order to eliminate the right-skewed distribution of green patent application data, we add 1 to the number of green patent applications and take the natural logarithm to obtain LnGI1, LnGI2, and LnGI3.

#### 4.2.2. Independent Variable

Digital transformation (*Digit_i,t−_*_1_) is the independent variable in this paper. We assign *Digit_i,t−_*_1_ based on the quasi-natural experiment of “Integration of Informatization and Industrialization” pilot. In 2014, the pilot project of the Integration of Informatization and Industrialization (IoII) was launched throughout China by the Ministry of Industry and Information Technology (MIIT). Pilot enterprises began to implement digital transformation in accordance with the Assessment Specification (GBT23020-2013). As it takes time for a company’s digital transformation to have an impact on the green innovation, Digit is taken here with a lag of one period. Specifically, digit is 1 if the firm becomes a pilot enterprise of “Integration of Informatization and Industrialization”, and 0 otherwise. Therefore, we mainly observe the regression coefficient *α*_1_ of *Digit_i,t−_*_1_ in model (1). If *α*_1_ is significantly positive, it indicates that the corporate digital transformation obviously promotes green innovation; if *α*_1_ is significantly negative, it indicates that corporate digital transformation significantly inhibits green innovation.

#### 4.2.3. Control Variables

With reference to prior research [2,67,68,69], the following control variables are selected: firm size (Size), financial leverage (Lev), cash flow (Cfo), return on assets (ROA), growth rate (Growth), market power (Market), intensity of physical assets (PPE), listed years (LnAge), operating income (Income), year dummy variable (Year), industry dummy variable (Industry), and firm dummy variable (Firm).

The variables are defined and constructed in Table 1.

### 4.3. Sample and Data

As the scope of the pilot project “Integration of Informatization and Industrialization” is limited to manufacturing firms, the A-share listed manufacturing firms in Shanghai and Shenzhen Stock Exchanges are chosen as the sample. The firms that entered the pilot list are the treatment group, and the other firms are the control group. The new Accounting Standards for Enterprises in China went into effect on 1 January 2007, which is significantly different from the previous standards. Thus, the year 2007 is selected as the starting point for this study, and the sample span is chosen from 2007 to 2020. In total, 18,337 firm-year observations were obtained from 2230 firms from 2007 to 2020.

WIPO provides the most inclusive definition of green innovation, as well as the “International Patent Classification Green List”. To gain the number of green patents filed by sample firms each year, we obtain the patent classification number information for all A-share listed companies from the Chinese Research Data Services (CNRDS) and match it with the “Green List of International Patent Classification” issued by the WIPO.

The data of the pilot project of “Integration of Informatization and Industrialization” come from the pilot list published by the Ministry of Industry and Information Technology. Control variable data are from the CSMAR database. In addition, White’s heteroskedasticity test and Robust’s robust standard error correction are applied to the multiple regressions, firm-level clustering (Cluster) is performed, and all continuous variables are winsorized at the upper and lower 1% levels.

## 5. Empirical Testing

### 5.1. Descriptive Statistics

The descriptive statistics for the variables are presented in Table 2. LnGI1 spans from 0 to 3.714, with a mean value of 0.416, indicating a substantial range in green innovation across listed manufacturing firms. According to the fact that the median of LnGI1, LnGI2, and LnGI3 all turn out to be 0, more than half of the listed manufacturing firms do not produce any green innovation output, suggesting that examining the influencing factors of green innovation in listed manufacturing firms has practical value. The average value of Digit is 0.071, signifying that the sample of pilot firms accounts for about 7.1% of the total sample.

**Figure 2:**
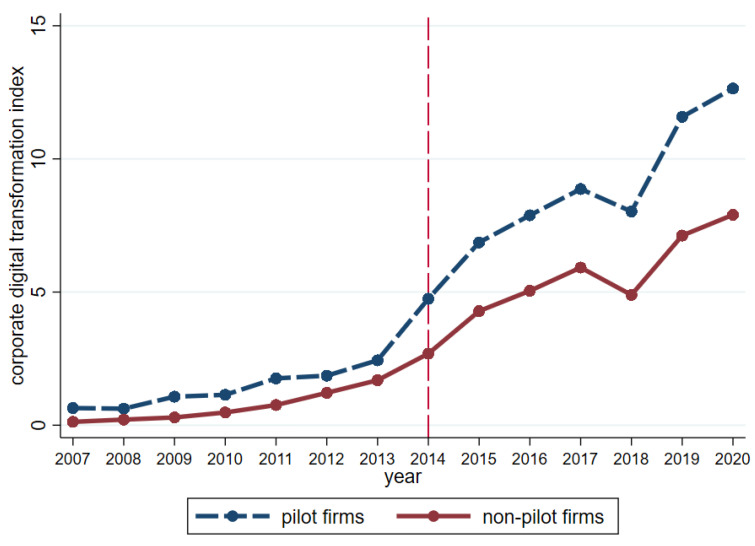
Digital transformation index of pilot firms and non-pilot firms.

In order to visually show whether the pilot project of “Integration of Informatization and Industrialization” can be used to represent corporate digital transformation, this paper uses the text analysis method to construct the corporate digital transformation index. We use Python crawler to collect a 2007–2020 annual report of listed companies, and then, we counted the disclosure times of keywords from five aspects: artificial intelligence technology, big data technology, cloud computing technology, blockchain technology, and digital technology application. We use the disclosure times excluding negative expressions and non-enterprise expressions to reflect the degree of corporate digital transformation. Finally, the word frequency data is standardized to obtain the corporate digital transformation index. Descriptive statistics are shown in Figure 2 for the digital transformation index which distinguishes pilot firms from non-pilot firms. The vertical dashed line in Figure 1 is the time when the pilot policy began to be implemented. It can be seen that before the implementation of the pilot policy of “Integration of Informatization and Industrialization”, the difference in digital transformation index between pilot firms from non-pilot firms remains unchanged, while after the implementation of the policy, the digital transformation index of pilot firms increases more significantly than non-pilot firms. It indicates that the pilot policy of “Integration of Informatization and Industrialization” has brought about the improvement of the digital level of pilot firms and shows that the pilot policy provides a good quasi-natural experimental scene for examining the economic consequences of corporate digital transformation.

### 5.2. Primary Results

Table 3 reports the regression analysis results for Model (1). The regression coefficients on Digit to LnGI1, LnGI2, and LnGI3 are 0.149, 0.131, and 0.097, respectively, all being substantially positive at the 1% level. This shows that with the improvement of corporate digitalization, the corporate green innovation output (LnGI1) increases. Moreover, corporate digitalization improves not only the quantity (LnGI3) but also the quality (LnGI2) of green innovation. The above statistics support Hypothesis 1.

In terms of control variables, the regression coefficients of Size are all significantly positive at the level of 5%, indicating that the larger the firm size is, the more the green innovation output is, and the corporate green innovation has an obvious scale effect. The regression coefficient of ROA is significantly positive at least at the level of 5%, suggesting that the higher the level of corporate profitability is, the more green innovation output is, primarily due to the endogenous financing provided by profitability which promotes green innovation investment. However, the regression coefficients of Growth are all significantly negative at the level of 1%, announcing that the faster a firm grows, the less green innovation output it produces.

### 5.3. Robustness Tests

#### 5.3.1. Parallel Trend Test

An important premise of the DID test is that the treatment and control groups have parallel trends prior to external policy shocks. Referencing Beck et al. [65], we use dummy variables such as three years prior to the list, two years prior to the list, one year prior to the list, the year of the list, and one year after the list to compare the green innovation output of treatment and control groups and to determine the differences in trends between the two groups. Table 4 illustrates that over the three years prior to the “Integration of Informatization and Industrialization“ (3 years before, 2 years before, and 1 year before), there are no significant differences between pilot firms and non-pilot firms in terms of green innovation output. Nonetheless, after the start of the integration (Year of list and 1 year after), the green innovation output of the pilot firms is significantly higher than that of the non-pilot firms. The above mentioned demonstrates that the DID test satisfies the premise assumption of parallel trend, thereby validating the preceding conclusion.

#### 5.3.2. PSM-DID

In order to eliminate the influence of the sample self-selection problem, we adopt the Propensity Score Matching (PSM) method according to Chen et al. [70] and construct a group of samples closest to the treatment group before policy implementation as the new control group. We take the pilot firms as the treatment group and find the most similar paired samples with the treatment group among the sample firms that have never been selected into the pilot list. The treatment and control groups are matched using the 1:1 proximity matching method. Table 5 shows the covariate balancing test results for each variable before and after matching. All the variables matching succeeds in making the means of the covariates close to each other for the treated and controls.

Table 6 displays the results of the PSM-DID test. The results indicate that the coefficient of Digit is significantly positive. This shows that with the improvement of corporate digitalization, the corporate green innovation output increases. The aforementioned findings reveal that even after controlling for the self-selection problem, the paper’s conclusions remain the same.

#### 5.3.3. Placebo Tests

**Figure 3:**
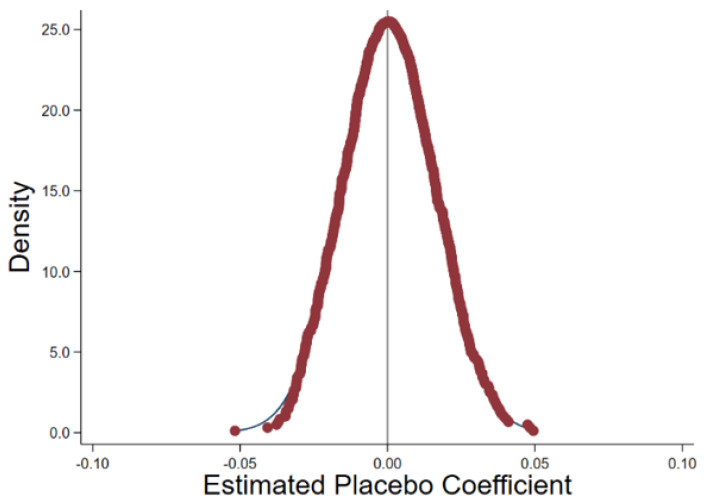
Distribution of placebo estimates.

In order to test the extent to which the above results are affected by omitted variables and random factors, with reference to Chetty et al. [71], Li et al. [72], and Cantoni et al. [73], we construct placebo tests by randomly selecting pilot firms and repeating this process 1000 times. Figure 3 shows that the regression coefficients from the stochastic simulation are distributed around 0, while the coefficients 0.149 from the benchmark regression are completely independent of this coefficient distribution, indicating that the empirical results are not caused by omitted variables and random factors.

#### 5.3.4. Delete Observations That Entered the Pilot List in the Current Year

Since firms may react early in anticipation before the launch of the pilot policy and may overreact afterwards, robustness tests are conducted by excluding observations of the year when firms enter into the pilot list. Table 7 displays results of regression. The regression coefficients for Digit are all significantly positive at the 1% level, consistent with the results of the benchmark regression. This shows that after excluding the observations in the year of entering the pilot list, the conclusion of this paper remains unchanged.

## 6. Extended Analyses

In the primary test, this paper studies the impact of corporate digital transformation on green innovation from a full-sample viewpoint. The results demonstrate that corporate digital transformation can greatly boost green innovation output. Under varied external circumstances and enterprise attributes, digital transformation’s impact on green innovation may be asymmetric. Thus, we divide the entire sample into groups based on the level of regional digital economy environment, the degree of competition in the industry and the firm size to implement extended analyses.

### 6.1. The Impact of the Regional Digital Economy Environment

With reference to Tan et al. [74], we use The White Paper on Digital Economy Index of Chinese Cities to measure the regional digital economy environment, classifying the top 30 cities as the high group and the remaining cities as the low group. The results of the grouping empirical test are displayed in Table 8. In the group with a high level of regional digital economy, the coefficients on Digit are significantly positive at the 1% level, whereas they are not significantly positive in the group with a low level of regional digital economy. The coefficients of Digit are significantly different between groups according to empirical *p*-values. The empirical results suggest that corporate digital transformation promotes green innovation output more in regions with an ailing digital economy. This can be explained by the fact that in regions with a low level of digital economy environment, corporate green innovation output is low, so corporate digital transformation can play a more effective role and significantly improve the corporate green innovation output. However, in regions with a high level of digital economy environment, corporate green innovation output is high, thus the promotion effect of digital transformation on their green innovation output is relatively insignificant.

### 6.2. Impact of Industrial Competition

We examine whether the impact of digital transformation on green innovation varies according to the degree of industrial competition. Our proxy for industrial competition is the Herfindahl–Hirschman Index (HHI), a widely used measure of market concentration. HHI is inversely related to industrial competition. The index is calculated as the sum of squares of market shares in an industry. Using the sales data from CSMAR, we measure a firm’s market share as the ratio of the firm’s sales to the sum of sales of all firms in the industry. HHI ranges from 0 to 1, moving from a large number of very small firms (i.e., high competition industry) to a monopolistic producer (i.e., low competition industry).

We divide the entire samples into groups according to the median of industry competition degree and the results are shown in Table 9. The coefficients of Digit are significantly positive at the 1% level when industrial competitiveness is low but insignificant when it is high. The coefficients of Digit differ significantly between groups in line with empirical *p*-values. The empirical results show that digital transformation has a more significant impact on corporate green innovation with a low degree of competition compared to those with a high degree of competition. Grossman and Helpman [75] discovered that the following effect and imitation behavior caused by high market competition will weaken the increment of unit product value generated by innovation, thus inhibiting the innovation motivation of management. In industries with high competition, the value of green innovation output is inherently low, and firms are not sufficiently motivated to innovate, making it difficult for digital transformation to reverse firms’ willingness to innovate; whereas in industries with low competition, the value of green innovation output is inherently high, thus firms are more motivated to innovate. Therefore, the effect of digital transformation on green innovations output is considerably more pronounced in industries with low competition than in those with high competition.

### 6.3. Impact of Firm Size

In order to assess the influence of corporate digital transformation on green innovation in firms of various sizes, the sample firms are divided into two groups based on the median of size. The results are presented in Table 10. In big firms, Digit’s coefficients are significantly positive at the 1% level, but not in small firms. The coefficients of Digit differ significantly between groups according to the empirical *p*-values. The empirical results indicate that digital transformation increases more output of green innovation in larger firms than in smaller ones. In the process of digital transformation, firms optimize their internal environment, enhance their risk assessment capabilities, and improve their information and communication efficiency, hence increasing green innovation output. The development of digital economy further strengthens the form of network economy, which makes the social network show obvious network externalities. If the digital economy mainly produces positive network externalities, the Matthew Effect will occur, generating self-expansion of the consumption scale and the scaling effect. Digital technologies require large-scale collaboration and real-time connections to be effective [76]. When the production, sales, and other links of enterprises reach a certain critical value, digital transformation will trigger positive feedback. Therefore, digital transformation is more likely to boost green innovation when the firm scale is larger.

## 7. Conclusions

This paper studies the economic consequences of corporate digital transformation via the lens of green innovation. Using a quasi-natural experimental scenario of the gradual deployment of “Integration of Informatization and Industrialization”, pilot firms are the treatment group and non-pilot firms are the control group. A DID model is used to evaluate the causal relationship and value enhancement mechanism between corporate digital transformation and green innovation, as well as how regional digital economy environment, industry competition, and firm size influence this relationship. The following conclusions are drawn.

Green innovation output significantly increases after firms become pilots of the “Integration of Informatization and Industrialization” project, and the influence of corporate digital transformation on green innovation output is more substantial in regions with ailing digital economy, industries with lesser competition, and firms with larger size. It signifies that corporate digital transformation can boost green innovation output, but it is susceptible to the macro external environment, meso industry characteristics, and micro firm features. To evaluate the robustness of the empirical results, a parallel trend test, PSM-DID, Placebo tests, and delete observations which entered the pilot list in the current year are used, and the conclusion remains the same.

As the Integration of Informatization and Industrialization is an essential endeavor to promote corporate digital transformation and digital economy in China, its implementation effect has significant implications for China’s industrial transformation and upgrade, as well as the further development of digital industrialization and industrial digitization.

Nevertheless, this paper still has several limitations. First, due to the pilot policy, the sample firms in this study are confined to manufacturing industry, thus additional research is required to determine whether the findings are applicable to other industries. Then, the potential mechanisms by which corporate digital transformation affects green innovation need to be further explored. In addition, previous studies on the economic consequences of digital transformation still lack empirical evidence from the standpoint of micro enterprises, and future research can shed more light on this field.

## Figures and Tables

**Table 1:** Variable definitions.

Variable	Explanation	Definition
LnGI1	Green innovation	Natural logarithm of 1 plus the aggregate number of green patents filed in application
LnGI2	Green innovation	Natural logarithm of 1 plus the aggregate number of green invention patents filed in application
LnGI3	Green innovation	Natural logarithm of 1 plus the aggregate number of green utility patents filed in application
Digit	Digital transformation	A dummy variable that equals one after the firm enters the pilot list for Integration of Informatization and Industrialization, and zero otherwise
Size	Firm size	The natural logarithm of total assets at the fiscal year end
Lev	Financial leverage	The ratio of total liabilities to total assets
Cfo	Cash flow	The ratio of net cash flow from operations to total assetstotal assets
ROA	Return on assets	The ratio of net income to total assets
Growth	Growth rate	The ratio of operating income change to operating income in the previous period at every year end
Market	Market power	Natural logarithm of the ratio of operating income to operating cost
PPE	Intensity of physical assets	The ratio of net property, plant, and equipment tototal assets
LnAge	Listed years	The natural logarithm of the firm’s listed years plus one
Income	Operating income	Natural logarithm of 1 plus the operating income
Year	Year dummy variable	Year fixed effects
Industry	Industry dummy variable	Industry fixed effects, based on guidelines for industry category of Chinese listed firms in 2012
Firm	Firm dummy variable	Firm fixed effects

**Table 2:** Summary statistics.

Variable	Obs.	Mean	S.D.	Minimum	Median	Maximum
LnGI1	18,337	0.416	0.815	0.000	0.000	3.714
LnGI2	18,337	0.272	0.637	0.000	0.000	3.219
LnGI3	18,337	0.255	0.596	0.000	0.000	2.890
Digit	18,337	0.071	0.256	0.000	0.000	1.000
Size	18,337	21.956	1.161	19.712	21.812	25.419
Lev	18,337	0.420	0.205	0.056	0.410	0.988
Cfo	18,337	0.050	0.068	−0.148	0.048	0.244
ROA	18,337	0.036	0.071	−0.327	0.037	0.211
Growth	18,337	0.160	0.368	−0.519	0.106	2.287
Market	18,337	0.354	0.296	−0.023	0.276	1.629
PPE	18,337	0.246	0.141	0.019	0.220	0.643
LnAge	18,337	2.797	0.339	1.792	2.833	3.434
Income	18,337	21.375	1.352	18.367	21.250	25.161

**Table 3:** Corporate digital transformation and green innovation.

	(1)	(2)	(2)
	LnGI1	LnGI2	LnGI3
Digit	0.149 ***	0.131 ***	0.097 ***
	(3.428)	(3.379)	(3.037)
Size	0.064 ***	0.056 ***	0.042 **
	(2.677)	(2.963)	(2.304)
Lev	0.030	0.043	0.009
	(0.594)	(1.036)	(0.239)
Cfo	−0.019	0.022	−0.070
	(−0.243)	(0.333)	(−1.170)
ROA	0.215 ***	0.180 ***	0.131 **
	(2.661)	(2.797)	(2.106)
Growth	−0.053 ***	−0.048 ***	−0.031 ***
	(−4.376)	(−4.803)	(−3.430)
Market	−0.052	−0.052	−0.024
	(−1.129)	(−1.641)	(−0.653)
PPE	−0.012	−0.045	0.045
	(−0.169)	(−0.804)	(0.873)
LnAge	0.081	0.060	0.075
	(0.617)	(0.583)	(0.692)
Income	0.013	0.009	0.006
	(0.647)	(0.569)	(0.446)
_cons	−1.603 ***	−1.353 ***	−1.082 ***
	(−3.580)	(−3.990)	(−2.925)
Year	Yes	Yes	Yes
Industry	Yes	Yes	Yes
Firm	Yes	Yes	Yes
N	18,337	18,337	18,337
Adj.R^2^	0.047	0.044	0.037

Note: Standard errors in parentheses. ** and *** indicate statistical significance at the 5% and 1% levels, respectively.

**Table 4:** The results of the parallel trend test.

	(1)	(2)	(2)
	LnGI1	LnGI2	LnGI3
3 years before	−0.067	−0.139	0.011
	(−0.901)	(−1.084)	(0.171)
2 years before	−0.045	−0.107	0.024
	(−0.792)	(−1.255)	(0.494)
1 year before	−0.012	−0.049	0.014
	(−0.201)	(−0.829)	(0.311)
Year of list	0.159 *	0.001	0.147 *
	(1.724)	(0.010)	(1.738)
1 year after	0.203 ***	0.110 *	0.149 ***
	(3.091)	(1.776)	(3.017)
Size	0.063 ***	0.280 ***	0.041 **
	(2.644)	(9.252)	(2.294)
Lev	0.027	−0.067	0.008
	(0.518)	(−1.054)	(0.213)
Cfo	−0.021	0.015	−0.071
	(−0.264)	(0.176)	(−1.178)
ROA	0.216 ***	0.174 *	0.132 **
	(2.678)	(1.773)	(2.132)
Growth	−0.052 ***	−0.048 ***	−0.031 ***
	(−4.338)	(−2.946)	(−3.386)
Market	−0.053	−0.195 ***	−0.024
	(−1.154)	(−3.815)	(−0.662)
PPE	−0.011	−0.049	0.048
	(−0.151)	(−0.594)	(0.924)
LnAge	0.076	−0.086	0.074
	(0.577)	(−0.617)	(0.681)
Income	0.014	0.026	0.007
	(0.696)	(1.109)	(0.475)
_cons	−1.562 ***	−6.264 ***	−1.073 ***
	(−3.492)	(−11.568)	(−2.898)
Year	Yes	Yes	Yes
Industry	Yes	Yes	Yes
Firm	Yes	Yes	Yes
N	18,337	18,337	18,337
Adj.R^2^	0.048	0.187	0.038

Note: Standard errors in parentheses. *, **, and *** indicate statistical significance at the 10%, 5%, and 1% levels, respectively.

**Table 5:** Test of covariate balancing.

Variable	Unmatched Matched	Mean		%Bias	%Reduct |Bias|	*t*-Test	
		Treated	Control			*t*	*p* > |*t*|
Size	U	22.372	21.903	42.1		5.66	0.000
	M	22.372	22.372	0	100	0.00	0.998
Lev	U	0.436	0.413	11.4		1.54	0.123
	M	0.436	0.433	1.5	87.1	0.15	0.882
Cfo	U	0.058	0.049	14.4		1.91	0.056
	M	0.058	0.055	4.4	69.6	0.42	0.676
ROA	U	0.049	0.034	23.4		2.81	0.005
	M	0.049	0.050	−1.8	92.4	−0.21	0.838
Growth	U	0.145	0.155	−2.9		−0.35	0.727
	M	0.145	0.162	−5.3	−82.6	−0.53	0.595
Market	U	0.394	0.353	13.6		1.94	0.053
	M	0.394	0.401	−2.2	83.9	−0.20	0.842
PPE	U	0.256	0.243	10		1.36	0.175
	M	0.256	0.261	−3.4	65.9	−0.33	0.744
LnAge	U	2.785	2.810	−8.2		−1.06	0.291
	M	2.785	2.773	3.8	53.2	0.38	0.704
Income	U	21.796	21.304	38.2		5.08	0.000
	M	21.796	21.770	2	94.7	0.19	0.848

**Table 6:** PSM-DID.

	(1)	(2)	(2)
	LnGI1	LnGI2	LnGI3
Digit	0.128 **	0.116 ***	0.084 **
	(2.429)	(2.594)	(2.127)
Size	0.028	0.035	0.026
	(0.414)	(0.659)	(0.520)
Lev	−0.018	0.005	−0.015
	(−0.139)	(0.045)	(−0.161)
Cfo	−0.028	0.048	−0.130
	(−0.162)	(0.322)	(−1.009)
ROA	0.467 *	0.381 *	0.279
	(1.796)	(1.810)	(1.426)
Growth	−0.103 ***	−0.092 ***	−0.053 **
	(−3.313)	(−3.653)	(−2.160)
Market	−0.189	−0.183 **	−0.121
	(−1.468)	(−1.983)	(−1.259)
PPE	−0.165	−0.165	−0.002
	(−0.918)	(−1.150)	(−0.013)
LnAge	0.161	0.178	0.073
	(0.554)	(0.693)	(0.355)
Income	0.061	0.064	0.017
	(1.044)	(1.266)	(0.417)
_cons	−2.046 *	−2.218 **	−1.162
	(−1.765)	(−2.259)	(−1.421)
Year	Yes	Yes	Yes
Industry	Yes	Yes	Yes
Firm	Yes	Yes	Yes
N	4221	4221	4221
Adj.R^2^	0.073	0.071	0.051

Note: Standard errors in parentheses. *, **, and *** indicate statistical significance at the 10%, 5%, and 1% levels, respectively.

**Table 7:** The results after deleting the current year of the list sample.

	(1)	(2)	(2)
	LnGI1	LnGI2	LnGI3
Digit	0.142 ***	0.127 ***	0.093 ***
	(2.969)	(3.038)	(2.635)
Size	0.064 ***	0.055 ***	0.041 **
	(2.656)	(2.929)	(2.278)
Lev	0.034	0.048	0.007
	(0.655)	(1.171)	(0.191)
Cfo	−0.013	0.027	−0.067
	(−0.170)	(0.415)	(−1.102)
ROA	0.213 ***	0.180 ***	0.131 **
	(2.634)	(2.799)	(2.113)
Growth	−0.052 ***	−0.048 ***	−0.031 ***
	(−4.352)	(−4.803)	(−3.462)
Market	−0.054	−0.055 *	−0.024
	(−1.169)	(−1.740)	(−0.650)
PPE	−0.007	−0.042	0.047
	(−0.102)	(−0.751)	(0.916)
LnAge	0.074	0.054	0.076
	(0.562)	(0.523)	(0.696)
Income	0.012	0.008	0.006
	(0.606)	(0.508)	(0.426)
_cons	−1.567 ***	−1.311 ***	−1.070 ***
	(−3.500)	(−3.881)	(−2.889)
Year	Yes	Yes	Yes
Industry	Yes	Yes	Yes
Firm	Yes	Yes	Yes
N	18,129	18,129	18,129
Adj.R^2^	0.046	0.043	0.036

Note: Standard errors in parentheses. *, **, and *** indicate statistical significance at the 10%, 5%, and 1% levels, respectively.

**Table 8:** Heterogeneity test for regional digital environment.

	LnGI1		LnGI2		LnGI3	
	(1)	(2)	(3)	(4)	(5)	(6)
	High	Low	High	Low	High	Low
Digit	0.089	0.205 ***	0.092	0.170 ***	0.069	0.121 ***
	(1.425)	(3.451)	(1.535)	(3.310)	(1.496)	(2.738)
Size	0.114 ***	0.051 *	0.110 ***	0.032	0.067 **	0.039 *
	(3.024)	(1.685)	(3.460)	(1.363)	(2.310)	(1.717)
Lev	0.015	−0.039	0.001	0.027	0.014	−0.049
	(0.181)	(−0.606)	(0.010)	(0.583)	(0.244)	(−1.009)
Cfo	0.048	−0.097	0.074	−0.019	−0.080	−0.089
	(0.402)	(−0.944)	(0.733)	(−0.220)	(−0.829)	(−1.157)
ROA	0.243 **	0.142	0.256 ***	0.101	0.075	0.134
	(2.109)	(1.272)	(2.735)	(1.140)	(0.823)	(1.597)
Growth	−0.039 **	−0.068 ***	−0.050 ***	−0.044 ***	−0.018	−0.048 ***
	(−2.205)	(−4.237)	(−3.116)	(−3.660)	(−1.402)	(−3.828)
Market	0.017	−0.068	−0.007	−0.054	0.024	−0.042
	(0.279)	(−0.945)	(−0.133)	(−1.264)	(0.527)	(−0.722)
PPE	0.036	−0.028	0.022	−0.085	0.013	0.083
	(0.337)	(−0.295)	(0.243)	(−1.159)	(0.157)	(1.163)
LnAge	0.143	−0.053	0.079	0.008	0.159	−0.065
	(0.656)	(−0.373)	(0.462)	(0.066)	(0.845)	(−0.641)
Income	0.019	0.009	0.009	0.006	0.016	0.001
	(0.628)	(0.343)	(0.365)	(0.321)	(0.708)	(0.036)
_cons	−2.806 ***	−0.933 *	−2.502 ***	−0.706 *	−1.903 ***	−0.591
	(−3.942)	(−1.696)	(−4.346)	(−1.685)	(−3.197)	(−1.368)
Firm	Yes	Yes	Yes	Yes	Yes	Yes
Year	Yes	Yes	Yes	Yes	Yes	Yes
Industry	Yes	Yes	Yes	Yes	Yes	Yes
N	9298	9039	9298	9039	9298	9039
Adj.R^2^	0.048	0.056	0.047	0.049	0.038	0.042
Empirical *p*-values	0.044 ***		0.023 ***		0.102 ***	

Note: Standard errors in parentheses. *, **, and *** indicate statistical significance at the 10%, 5%, and 1% levels, respectively. Empirical *p*-values are used to test the significance of the between-group differences in Digit coefficients.

**Table 9:** Heterogeneity test for the industry competition.

	LnGI1		LnGI2		LnGI3	
	(1)	(2)	(3)	(4)	(5)	(6)
	Low	High	Low	High	Low	High
Digit	0.162 ***	0.060	0.136 ***	0.070	0.116 ***	0.012
	(3.036)	(1.090)	(2.853)	(1.476)	(2.841)	(0.329)
Size	0.060 *	0.011	0.056 **	0.024	0.041	−0.015
	(1.824)	(0.343)	(2.235)	(0.985)	(1.594)	(−0.611)
Lev	−0.010	0.010	−0.001	0.016	0.017	−0.024
	(−0.145)	(0.129)	(−0.018)	(0.275)	(0.321)	(−0.474)
Cfo	−0.042	−0.006	−0.019	0.079	−0.088	−0.027
	(−0.424)	(−0.048)	(−0.233)	(0.883)	(−1.127)	(−0.283)
ROA	0.165	0.236 *	0.159 *	0.131	0.117	0.103
	(1.590)	(1.922)	(1.940)	(1.398)	(1.418)	(1.112)
Growth	−0.068 ***	−0.019	−0.058 ***	−0.019	−0.042 ***	−0.007
	(−4.579)	(−0.852)	(−4.636)	(−1.124)	(−3.645)	(−0.466)
Market	0.003	0.002	−0.019	0.010	0.022	−0.005
	(0.042)	(0.041)	(−0.378)	(0.230)	(0.386)	(−0.137)
PPE	−0.062	0.063	−0.088	0.042	0.027	0.049
	(−0.675)	(0.630)	(−1.238)	(0.488)	(0.359)	(0.789)
LnAge	0.097	−0.208	0.076	−0.156	0.070	−0.033
	(0.555)	(−1.215)	(0.554)	(−1.167)	(0.468)	(−0.328)
Income	0.033	0.030	0.021	0.016	0.021	0.018
	(1.277)	(0.963)	(1.010)	(0.681)	(1.119)	(0.835)
_cons	−1.817 ***	−0.309	−1.549 ***	−0.429	−1.259 **	−0.016
	(−2.989)	(−0.474)	(−3.368)	(−0.867)	(−2.424)	(−0.041)
Year	Yes	Yes	Yes	Yes	Yes	Yes
Industry	Yes	Yes	Yes	Yes	Yes	Yes
Firm	Yes	Yes	Yes	Yes	Yes	Yes
N	13,340	4997	13,340	4997	13,340	4997
Adj.R^2^	0.044	0.025	0.043	0.014	0.039	0.020
Empirical *p*-values	0.126 ***		0.115 ***		0.116 ***	

Note: Standard errors in parentheses. *, **, and *** indicate statistical significance at the 10%, 5%, and 1% levels, respectively. Empirical *p*-values are used to test the significance of the between-group differences in Digit coefficients.

**Table 10:** Heterogeneity test for firm size.

	LnGI1		LnGI2		LnGI3	
	(1)	(2)	(3)	(4)	(5)	(6)
	Big	Small	Big	Small	Big	Small
Digit	0.202 ***	0.057	0.171 ***	0.036	0.138 ***	0.024
	(3.397)	(1.177)	(3.153)	(0.881)	(3.005)	(0.700)
Size	0.105 *	0.071 ***	0.113 **	0.046 ***	0.053	0.041 **
	(1.908)	(2.766)	(2.556)	(2.605)	(1.234)	(2.063)
Lev	0.016	−0.057	0.051	−0.033	−0.027	−0.035
	(0.150)	(−1.168)	(0.562)	(−0.932)	(−0.336)	(−1.042)
Cfo	−0.067	−0.054	−0.016	−0.033	−0.107	−0.048
	(−0.514)	(−0.592)	(−0.140)	(−0.479)	(−1.066)	(−0.689)
ROA	0.434 **	−0.028	0.411 ***	−0.031	0.231 *	−0.026
	(2.406)	(−0.379)	(2.763)	(−0.561)	(1.673)	(−0.438)
Growth	−0.045 **	−0.033 **	−0.040 **	−0.023 *	−0.037 ***	−0.019
	(−2.485)	(−1.976)	(−2.556)	(−1.868)	(−2.646)	(−1.506)
Market	−0.014	0.013	−0.041	−0.005	0.015	0.019
	(−0.147)	(0.308)	(−0.517)	(−0.167)	(0.216)	(0.553)
PPE	−0.044	−0.028	−0.071	−0.090	0.035	0.052
	(−0.331)	(−0.351)	(−0.666)	(−1.577)	(0.348)	(0.885)
LnAge	−0.077	0.123	−0.038	0.185 **	−0.053	0.010
	(−0.285)	(0.999)	(−0.177)	(2.180)	(−0.225)	(0.117)
Income	0.007	0.030 *	−0.002	0.017	0.013	0.019
	(0.173)	(1.665)	(−0.072)	(1.381)	(0.470)	(1.436)
_cons	−1.887 *	−2.220 ***	−2.050 **	−1.650 ***	−1.137	−1.159 ***
	(−1.793)	(−4.588)	(−2.429)	(−4.877)	(−1.339)	(−3.096)
Year	Yes	Yes	Yes	Yes	Yes	Yes
Industry	Yes	Yes	Yes	Yes	Yes	Yes
Firm	Yes	Yes	Yes	Yes	Yes	Yes
N	9172	9165	9172	9165	9172	9165
Adj.R^2^	0.068	0.024	0.071	0.016	0.046	0.023
Empirical *p*-values	0.201 ***		0.158 ***		0.133 ***	

Note: Standard errors in parentheses. *, **, and *** indicate statistical significance at the 10%, 5%, and 1% levels, respectively. Empirical *p*-values are used to test the significance of the between-group differences in Digit coefficients.

## Data Availability

Not applicable.
